# A comprehensive overview of genetic mutations in Iranian patients with Bardet-Biedl syndrome

**DOI:** 10.1016/j.ymgmr.2026.101357

**Published:** 2026-09-15

**Authors:** Sheyda Khalilian, Mohadeseh Fathi, Mona Entezam, Zahra Farbood, Soudeh Ghafouri-Fard, Seyed Alireza Dastgheib, Mohammad Miryounesi

**Affiliations:** a Student Research Committee, School of Medicine, Shahid Beheshti University of Medical Sciences, Tehran, Iran; b Department of Medical genetics, Shahid Beheshti University of Medical Sciences, Tehran, Iran; c Department of Medical genetics, Shiraz University of Medical Sciences, Shiraz, Iran

**Keywords:** Bardet-Biedl syndrome, BBS, Ciliopathy, Whole-exome sequencing, Iranian population, BBS10, Consanguinity

## Abstract

**Background:**

Bardet-Biedl syndrome (BBS) is a rare, autosomal recessive ciliopathy characterized by pleiotropic clinical manifestations including retinal degeneration, obesity, polydactyly, intellectual disability, renal abnormalities, and hypogenitalism. Significant genetic heterogeneity exists, with over 20 genes implicated in its pathogenesis. This study provides a comprehensive overview of the genetic mutations identified in a cohort of Iranian patients with BBS.

**Methods:**

A total of 26 unrelated Iranian patients clinically suspected of BBS were enrolled. Whole-exome sequencing (WES) was performed on an Illumina HiSeq4000 platform.

**Results:**

Causative or potentially causative variants were identified in all 26 probands across 13 distinct genes. The most frequently mutated gene was *BBS10* (*n* = 3), followed by *BBS2* (*n* = 3), *BBS5* (*n* = 3), *BBS12* (*n* = 3), and *CEP290* (*n* = 3). Other affected genes included *BBS1* (*n* = 2), *SDCCAG8* (*n* = 2), and single cases involving *BBIP1*, *BBS7*, *TTC8*, *MKS1*, *IFT172*, *TRIM32*, and *GRID2*. All identified variants followed an autosomal recessive pattern, with 15 variants classified as pathogenic, 5 as likely pathogenic, and 7 as variants of uncertain significance (VUS). Consanguinity was present in 88.4% of families, consistent with the high rate of homozygous mutations observed. One patient (Case 5) exhibited a dual diagnosis with a homozygous pathogenic variant in *GJB2* associated with autosomal recessive deafness.

**Conclusion:**

This study expands the mutational spectrum of BBS in the Iranian population, highlighting the predominance of *BBS2*, *BBS5* and *BBS10* pathogenic/likely pathogenic variants. WES can provide a high diagnostic yield and represent an effective first-line or early diagnostic approach in clinically suspected genetically heterogeneous BBS/ciliopathy cases, particularly where appropriate CNV analysis is included. The high rate of consanguinity underscores the importance of homozygous variant detection. These findings facilitate accurate genetic counseling, enable carrier screening in at-risk families, and support the development of targeted molecular diagnostic strategies for BBS in Iran.

## 1 Introduction

Bardet–Biedl syndrome (BBS) is a human genetic condition inherited in an autosomal recessive pattern, affecting multiple body systems. While autosomal recessive inheritance is the primary mode of transmission in BBS, there have been rare cases of oligogenic or triallelic inheritance. However, these mechanisms are still debated and seem to represent only a small fraction of cases [1]. It is defined by six primary features: obesity, intellectual disability, kidney abnormalities, polydactyly, retinal degeneration, and hypogenitalism [2]. Additional minor symptoms, known as secondary features, may occur in some individuals with BBS but are not universally present. These include liver fibrosis, diabetes, neurological issues, speech and language difficulties, behavioral characteristics, poor coordination (ataxia), distinctive facial features, dental abnormalities, delayed development, high blood pressure, brachydactyly, syndactyly, heart defects, reproductive system abnormalities, hormone imbalances, short stature, and hearing loss [2]. According to the proposed diagnostic guidelines for BBS, a diagnosis requires either four major features or three major features along with two minor features—an approach that helps distinguish BBS from other syndromes with overlapping symptoms [3]. Nevertheless, depending solely on clinical symptoms may not be sufficient, making genetic testing necessary. In recent decades, the use of whole-exome sequencing (WES) has simplified the diagnosis of this condition.

BBS is regarded as a heterogeneous disorder stemming from mutations in various individual genes or genetic *loci*. Moreover, in certain BBS cases, interactions involving two or more *loci* have been reported. Most known BBS-related genes play a role in the formation and proper function of a multi-protein assembly known as the BBSome. This complex is made up of eight subunit proteins (BBS1, BBS2, BBS4, BBS5, BBS7, BBS8, BBS9, and BBS18), which are essential for normal ciliary function. The BBSome contributes to this, at least in part, through its interaction with Rab8's GTP/GDP exchange factor (Rabin8). BBS3/ARL6 facilitates the attachment of the BBSome to cellular membrane, while BBS17/LZTFL1 regulates its entrance into cilia. BBS6, BBS10, and BBS12 initially bind to and stabilize BBS7, resulting in the formation of an intermediate assembly called the BBSome core. In contrast to the BBSome, which is normally not necessary for the initial assembly of cilia, the intraflagellar transport (IFT) complex governs bidirectional movement along the axoneme, a process critical for the formation, maintenance, and function of cilia [4], [5]. Within the BBSome complex, a defect in any single subunit can result in the BBS phenotype. To gain access to cilia, the BBSome must pass through a regulatory barrier known as the transition zone, located at the ciliary base, which contains numerous protein complexes, including the Cep290/NPHP5 complex. This complex helps maintain the structural and functional integrity of the BBSome [6]. In addition to the BBSome, various other protein complexes are vital for proper ciliary function. Notably, the intraflagellar transport (IFT) machinery enables efficient bidirectional movement along the axoneme, while the transition zone complex plays a crucial role in regulating the entry of proteins into cilia [7]. Key genes such as CEP290, MKS1, and SDCCAG8 encode essential transition zone proteins, and IFT172 is crucial for the assembly of IFT particles [8]. When mutations occur in these genes, they can result in Bardet-Biedl syndrome (BBS) or related ciliopathies, underscoring the immense genetic diversity and complexity of these conditions [8]. Understanding this genetic landscape is crucial for advancing research and developing targeted therapies.

In this manuscript, we overview the genetic variants of Bardet-Biedl Syndrome (BBS) in Iranian patients with diverse clinical features. Iran has one of the highest rates of consanguineous marriage, ranging from 38% to 45% [9], which increases the prevalence of autosomal recessive disorders like BBS. Despite this, molecular data on BBS in Iran is limited.

This study provides a genetic overview of BBS in a large Iranian cohort, essential for genetic counseling, carrier screening, and targeted diagnostics. Our findings will enhance genetic counseling and improve the understanding of associated variants in this population.

## 2 Methods

### 2.1 Recruitment of cases

This study involved 26 unrelated Iranian individuals suspected of having BBS. Enrollment criteria included at least four major features or three major features plus two minor features, based on the diagnostic criteria by Beales et al. (1999) [3] and updated by Forsythe and Beales (2013) [10]. In atypical cases where patients did not meet the full Beales criteria, clinical suspicion of a ciliopathy was raised based on the presence of syndromic features overlapping with other ciliopathies, including: Encephalocele and polycystic kidney disease (Case 22), Cerebellar atrophy, nystagmus, and severe global developmental delay (Case 20), Hypotonia with a positive family history of similar phenotype (Case 18), Myopathy and muscular dystrophy with atypical BBS features (Case 25). These features are recognized as part of the broader ciliopathy spectrum, which includes Joubert syndrome, Meckel syndrome, and Senior-Løken syndrome, among others [11]. In such cases, the combination of one or more major BBS features (e.g., retinal degeneration, polydactyly) along with these additional ciliopathy-related findings prompted clinical suspicion of BBS or a related ciliopathy. This approach is supported by the understanding that BBS shares significant clinical and genetic overlap with other ciliary disorders, and that strict application of the Beales criteria may miss atypical or early-onset presentations [10]. These patients were subsequently classified as suspected BBS for the purposes of genetic investigation because:

1. They presented with at least one cardinal BBS feature (e.g., polydactyly, retinal degeneration, or renal anomalies),
2. They exhibited additional syndromic features suggestive of a ciliary defect,
3. The genetic testing strategy (WES) was designed to identify mutations in known BBS and ciliopathy-associated genes, and
4. Molecular findings confirmed pathogenic variants in established BBS/ciliopathy genes (e.g., *MKS1*, *CEP290*, *TRIM32*), validating the clinical suspicion.

Cases were assessed in the Comprehensive Medical Genetics Center, Shiraz, Iran during 2019–2024. Informed consent forms were signed by patients or their legal representatives. All methods were carried out in accordance with relevant guidelines and regulations. All experimental protocols were approved by ethical committee of Shiraz University of Medical Sciences (IR.SUMS.MED.REC.1399.325)**.**

### 2.2 Clinical evaluations

A total of 26 unrelated Iranian patients with suspected BBS were enrolled in this study. Clinical features were extracted retrospectively from medical records and information provided by referring clinicians rather than a standardized phenotypic assessment. The cohort consisted of 14 females, 10 males, and 2 cases with unreported sex (Cases 10 and 22). Ages ranged from 4 months to 48 years. Consanguinity was reported in 23 out of 26 cases (88.4%), highlighting a strong background of parental relatedness.

**Fig. 1:**
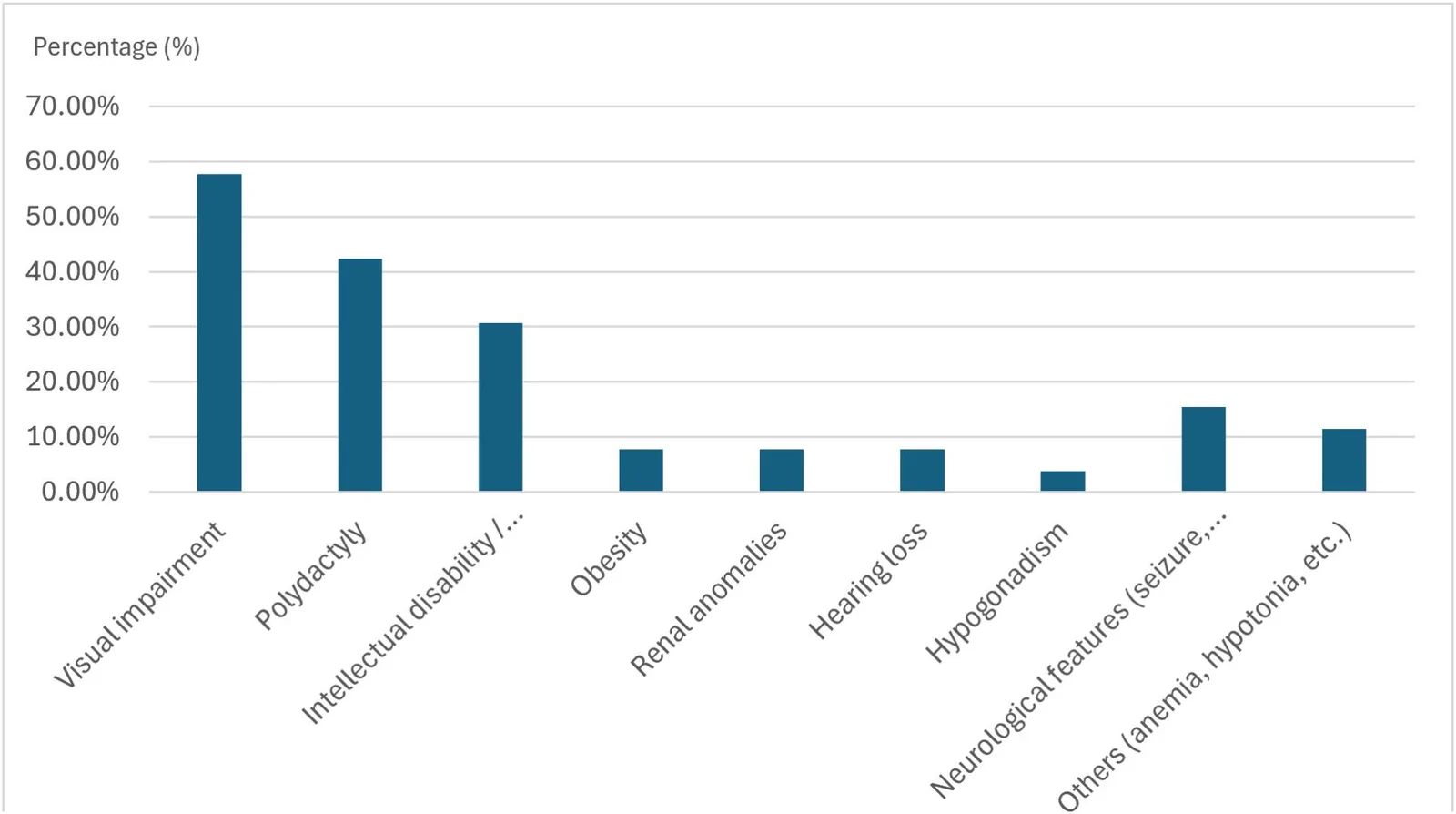
Prevalence of major and minor clinical features in 26 Iranian patients with Bardet–Biedl syndrome. ID/DD, intellectual disability/developmental delay; Hypogonadism includes micropenis and other forms; Renal anomalies include horseshoe kidney.

The most frequently observed clinical features were visual impairments, including retinopathy, retinitis pigmentosa (RP), night blindness, and progressive vision loss, which were present in most patients (e.g., Cases 1, 2, 6, 8, 9, 12, 14, 23 and 26). Polydactyly was another common major finding, documented in 11 out of 26 cases (42.3%). Intellectual disability or developmental delay was reported in 8 out of 26 cases (30.7%), including Cases 2, 4, 5, 15, 19, 20, 21, and 24. Obesity was noted in 2 out of 26 cases (7.7%). The frequency of clinical features is summarized in Fig. 1. Hypogonadism was explicitly reported as micropenis in Case 8. Additional neurological manifestations included seizures (Cases 11, 15 and 17), nystagmus (Cases 17 and 20), and cerebellar atrophy (Case 20). Other notable findings included hearing loss (Cases 5, 24), renal anomalies such as horseshoe kidney (Case 11) and polycystic kidney (Case 22), and musculoskeletal abnormalities including club foot (Case 10) and hypotonia (Case 18). Two cases (Cases 2 and 12) presented with anemia. Case 25 was notable for myopathy and muscular dystrophy, while Case 17 had elevated very long-chain fatty acids (VLCFA) and an abnormal EEG.

Family histories revealed additional complexities, including reports of diabetes, infertility, acute myeloid leukemia (AML), multiple sclerosis (MS), and similar phenotypes in siblings, particularly in Cases 3, 7, 13, and 20. Table 1 summarizes the clinical findings in these cases.

**Table 1:** Summary of clinical data of patients with Bardet-Biedl syndrome.

Case Number	Age	Sex	Consanguinity	Indications
1	27 Years	M	Yes	Retinopathy, night blindness, visual impairment, and hypothyroidism
2	28 Years	M	Yes	MR, blindness, obesity, polydactyly and anemia
3	21 Years	M	Yes	RP, blindness, developmental delay. There is history of blindness, diabetes, infertility, and AML in the family.
4	35 Years	M	Yes	MR, low vision, and polydactyly
5	30 Years	F	Yes	MR, night blindness, decreased vision, deafness and polydactyly
6	42 Years	M	No	Blindness, and polydactyly. There is a history of RP in the family.
7	23 Years	F	Yes	Vision problem, polydactyly, and suspicious of RP. There is history of diabetes type1 and MS in the family.
8	29 Years	M	Yes	Severe visual impairment, RP, MR, polydactyly, obesity, micropenis
9	34 Years	M	Yes	Visual impairment, photosensitivity, color blindness and RP
10	4 Months	–	Yes	Bilateral club foot, polydactyly, and brain cyst
11	27 Years	M	Yes	Severe low vision and night blindness, color blindness, polydactyly, horseshoe kidney and seizure
12	24 Years	F	Yes	RP, progressive visual impairment, visual loss, polydactyly, finger abnormality, and anemia
13	20 Years	F	Yes	Visual loss, night blindness. Her sister has the same phenotype.
14	28 Years	F	Yes	Low vision, color blindness, night blindness
15	7 Years	F	No	Hypoxia during birth, seizure, motor retardation and strabismus
16	35 Years	F	No	Polydactyly and visual impairment
17	3 Years	F	Yes	Seizure, nystagmus, head tremor, elevated VLCFA, and abnormal EEG
18	22 Months	M	Yes	Hypotonia, and similar family history
19	21 Years	F	Yes	MR, stuttering
20	15 Years	F	Yes	Global developmental delay, speech delay, MR, nystagmus, severe muscle weakness, and cerebellar atrophy. Her sister has the same symptoms.
21	9 Years	F	Yes	Developmental delay, MR, and speech defect
22	4 Months	–	Yes	Encephalocele and polycystic kidney
23	8 Years	F	Yes	Night vision problem, astigmatism
24	10 Months	F	Yes	Severe MR, lack of speech and hearing loss
25	48 Years	F	No	Myopathy and muscular dystrophy
26	21 Years	M	Yes	Night blindness, low vision, polydactyly and developmental delay

### 2.3 Molecular analyses

Genomic DNA was extracted from patient peripheral blood samples following the standard salting-out procedure. The purity and concentration of the DNA were measured using a NanoDrop 1000 spectrophotometer (Thermo Fisher Scientific, USA). WES was conducted on an Illumina HiSeq4000 platform, generating 101-base pair paired-end reads. Exonic regions and adjacent splice sites were enriched using the SureSelectXT2 V6 capture kit. After adjusting for capture efficiency and duplicate reads, the average sequencing coverage reached approximately 150×, with around 85% of target bases achieving at least 100× coverage. Raw sequencing data underwent quality control to eliminate low-quality reads, followed by alignment to the human reference genome (GRCh37/hg19) with the Burrows-Wheeler Aligner (BWA) [12]. Duplicate reads were identified and removed using SAMtools [13]. Base quality score recalibration and variant detection (SNPs and indels) were carried out using the Genome Analysis Toolkit (GATK v4) [14].

The variant filtering and prioritization strategy followed these steps: 1. Removed variants with a minor allele frequency (MAF) ≥ 0.01. 2. Retained variants in coding exons, splice sites (±10 bp), or untranslated regions. 3. Prioritized loss-of-function variants (nonsense, frameshift, splice-site) and missense variants with high pathogenicity scores (PolyPhen-2, SIFT, CADD). 4. Filtered for genes associated with Bardet-Biedl syndrome (BBS) or related ciliopathies. 5. Assessed zygosity and segregation with the autosomal recessive inheritance model. Finally, variants were functionally annotated and categorized with ANNOVAR [15]. Under an autosomal recessive inheritance model, allele read frequencies between 75 and 100% were considered for homozygous changes, whereas allele frequencies below 1% were used for recessive variants. Variant assessment was performed using VarSome, the Human Gene Mutation Database (HGMD) Professional 2019.4, and the dbSNP database. Minor allele frequency (MAF < 0.01) data were obtained from the Genome Aggregation Database v2.1.1 (gnomAD; https://gnomad.broadinstitute.org/). Then, gene variants were filtered and cross-referenced with several databases, such as dbSNP (https://www.ncbi.nlm.nih.gov/snp/), the 1000 Genomes Project (https://www.internationalgenome.org/), the Exome Variant Server (EVS; https://evs.gs.washington.edu/EVS/), and gnomAD (https://gnomad.broadinstitute.org/). In silico analysis was performed using several pathogenicity prediction tools: 1) PolyPhen-2 (http://genetics.bwh.harvard.edu/pph2/), 2) SIFT (https://sift.bii.a-star.edu.sg/), 3) VarSome (https://varsome.com/), and 4) CADD (https://cadd.gs.washington.edu/snv).

## 3 Results

WES and subsequent bioinformatic analysis identified potentially causal variants in BBS-related genes in nearly all probands (Table 2). A total of 13 distinct genes were implicated, including known *BBS* genes (*BBS1*, *BBS2*, *BBS5*, *BBS7*, *BBS10*, *BBS12*, *BBIP1*, *TTC8*, *CEP290*, *MKS1*, *SDCCAG8*, *TRIM32*, and *IFT172*) and one additional gene (*GRID2*) contributing to neurological features in a syndromic presentation. All variants were identified in a homozygous state except for two compound heterozygous variants in *CEP290* in Case 19.

**Table 2:** Summary of molecular data of patients with Bardet-Biedl syndrome.

Case number	Gene	Transcript	Variant	Associated disease	OMIM	Inheritance	Zygosity	ACMG classification	Clinvar	dbSNP rsID	Ref
1	*BBIP1*	NM_001195305.3	Exon 3 c.112 + 1G > A	Bardet-Biedl syndrome 18	615995	AR	Hom	Likely Pathogenic	NR	–	–
2	*BBS1*	NM_024649	Intron 4 c.432 + 1G > A	Bardet-Biedl syndrome 1	209900	AR	Hom	Pathogenic	Pathogenic	rs587777829	[16]
3	*BBS1*	NM_024649.5	Intron 11 c.1110 + 1G > C	Bardet-Biedl syndrome 1	209900	AR	Hom	Likely Pathogenic	Likely Pathogenic	–	–
4	*BBS2*	NM_031885.5	Intron 5 c.535-1G > C	Bardet-Biedl syndrome 2 Retinitis pigmentosa 74	615981 616562	AR AR	Hom	Pathogenic	Pathogenic	–	–
5	*BBS2*	–	deletion of exons 6–11 (5.4Kb)	Bardet-Biedl syndrome 2 Retinitis pigmentosa 74	615981 616562	AR	Hom	Pathogenic	–	–	
	*GJB2*	NM_004004.6	Exon 2 c.299_300delAT p.H100Rfs*14	Deafness, autosomal recessive 1 A	220290	AR	Hom	Pathogenic	–	rs111033204	–
6	*BBS2*	NM_031885.5	Intron 5 c.535-1G > C	Bardet-Biedl syndrome 2 Retinitis pigmentosa 74	615981 616562	AR	Hom	Pathogenic	Pathogenic	–	–
7	*BBS5*	NM_152384.3	Intron 8 c.681 + 1G > A	Bardet-Biedl syndrome 5	615983	AR	Hom	Likely Pathogenic	NR	–	–
8	*BBS5*	NM_152384.3	Exon 8 c.619-1G > C	Bardet-Biedl syndrome 5	615983	AR	Hom	Pathogenic	Pathogenic	rs753234582	–
9	*BBS5*	NM_152384.3	Exon 6 c.413G > A p.Arg138His	Bardet-Biedl syndrome 5	615983	AR	Hom	Pathogenic	Conflicting	rs179363897	[17]
10	*BBS7*	NM_176824.3	Exon 11 c.1083_1084del p.Asn362Leufs*21	Bardet-Biedl syndrome 7	615984	AR	Hom	Pathogenic	Conflicting	rs577434138	[18]
11	*BBS7*	NM_176824.3	Exon 19 c.2015G > T p.Gly672Val	Bardet-Biedl syndrome 7	615984	AR	Hom	VUS	VUS	–	–
12	*BBS10*	NM_024685.4	Exon 2 c.271dup p.Cys91Leufs*5	Bardet-Biedl syndrome 10	615987	AR	Hom	Pathogenic	Pathogenic	rs549625604	[19]
13	*BBS10*	NM_024685.4	Exon 2 c.1804G > T p.Val602Leu	Bardet-Biedl syndrome 10	615987	AR	Hom	Pathogenic	Likely Pathogenic	rs778431173	[20]
14	*BBS10*	NM_024685.4	Exon 2 c.1864delC p.H622Ifs*10	Bardet-Biedl syndrome 10	615987	AR	Hom	Likely Pathogenic	NR	–	–
15	*BBS12*	NM_152618.3	c.2024G > A p.Arg675Gln	Bardet-Biedl syndrome 12	615989	AR	Hom	VUS	VUS	rs144843913	–
16	*BBS12*	NM_152618.3	Exon 2 c.367 T > C p.Cys123Arg	Bardet-Biedl syndrome 12	615989	AR	Hom	VUS	Likely Pathogenic	–	[21]
17	*BBS12*	*NM_152618.3*	Exon 2 c.265_266del p.Leu89Valfs*11	Bardet-Biedl syndrome 12	615989	AR	Hom	Pathogenic	Conflicting	rs1397714772	–
18	*CEP290*	NM_025114.4	c. 5668G > T p.Gly1890*	Bardet-Biedl syndrome 14 Joubert syndrome 5 Meckel syndrome 4 Senior-Loken syndrome 6	615991 610188 611134 610189	AR	Hom	Pathogenic	Pathogenic	rs137852832	[22]
19	*CEP290*	*NM_025114.4*	Exon 43 c.5998 A > G p.Ile2000Val	Bardet-Biedl syndrome 14 Joubert syndrome 5 Meckel syndrome 4 Senior-Loken syndrome 6 Leber congenital amaurosis 10	615991 610188 611134 610189 611755	AR	Het	VUS	VUS	rs183071230	–
			Exon 12 c.963 T > A p.Asp321Glu			AR	Het	VUS	VUS	rs774072453	–
20	*CEP290*	NM_025114.4	Exon 50 c.6869dupA p.Asn2290Lysfs*6	Bardet-Biedl syndrome 14	615991 610188 611755 611134 610189	AR	Patient: Hom Parents: Het	Pathogenic	Pathogenic	rs587783017	–
	*GRID2*	NM_001510	Exon 11 c.1707_1713del p.Trp570Alafs*16	Spinocerebellar ataxia, autosomal recessive 18	616204	AR	Patient: Hom Parents: Het	Likely Pathogenic	–	–	–
21	*IFT172*	NM_015662.3	Exon 42 c.4612G > A p.Ala1538Thr	Bardet-Biedl syndrome 20 Retinitis pigmentosa 71 Short-rib thoracic dysplasia 10 with or without polydactyly	619471 616394 615630	AR	Hom	VUS	VUS	rs770733075	–
22	*MKS1*	NM_017777.4	Intron 5 c.515 + 1G > A	Meckel syndrome 1 Joubert syndrome 28 Bardet-Biedl syndrome 13	249000 617121 615990	AR	Hom	Pathogenic	Conflicting	rs201933838	[23]
23	*SDCCAG8*	NM_006642.5	Exon 12 c.1420del p.Glu474Serfs*20	Bardet-Biedl syndrome 16 Senior-Loken syndrome 7	615993 613615	AR	Hom	Pathogenic	Pathogenic	rs397515335	[24]
24	*SDCCAG8*	NM_006642.5	Exon 15 c.1489 T > G p.Phe595Val	Bardet-Biedl syndrome 16Senior-Loken syndrome 7	615,993,613,615	AR	Hom	VUS	Conflicting	rs776765317	–
25	*TRIM32*	NM_012210.4	Exon 2 c.1202_1203insGGAA p.Gly402Glufs*15	Bardet-Biedl syndrome 11 Muscular dystrophy, limb-girdle, autosomal recessive 8	615988 254110	AR	Hom	Likely Pathogenic	NR	–	–
26	*TTC8*	NM_144596.4	Exon 8 c.582_585del p.Phe196Serfs*56	Retinitis pigmentosa 51 Bardet-Biedl syndrome 8	613464615985	AR	Hom	Pathogenic	Conflicting	–	[25]

AR: autosomal recessive, Hom: homozygote, Het: heterozygote, VUS: variant of uncertain significance, NR: not reported.

Among the most frequently mutated gene was *BBS10*, identified in three cases (Cases 12, 13 and 14), all with homozygous pathogenic or likely pathogenic variants, including c.271dup (p.Cys91Leufs*5), c.1804G* *>* *T (p.Val602Leu), and c.1864delC (p.H622Ifs*10)*. BBS2* variants were also found in three cases (Cases 4, 5 and 6), including a recurrent splice site variant (c.535-1G > C) in Cases 4 and 6, and a large deletion of exons 6–11 in Case 5. Case 5 was a 30-year-old female diagnosed with BBS due to intellectual disability, night blindness, decreased vision, and polydactyly. She also had congenital profound sensorineural hearing loss, which is unusual for BBS. WES revealed a homozygous deletion in the *BBS2* gene (exons 6–11) and a homozygous variant in the *GJB2* gene (c.299_300delAT, p.H100Rfs14), known to cause autosomal recessive deafness. This combination of findings supports a dual molecular diagnosis for the patient. Variants in *BBS5* and *BBS12* were identified in three cases, respectively. In *BBS5*, the variants included c.681 + 1G > A, c.619-1G > C, and c.413G > A (p.Arg138His). In *BBS12*, variants included c.2024G > A (p.Arg675Gln), c.367 T > C (p.Cys123Arg) and c.265_266del (p.Leu89Valfs*11).

Pathogenic variants in *CEP290* were identified in three cases (Cases 18, 19 and 20). Case 18 carried a homozygous nonsense variant c.5668G > T (p.Gly1890*), and Case 20 carried a homozygous frameshift variant c.6869dupA (p.Asn2290Lysfs*6*)*. Case 20 might represent a dual diagnosis, since she had an additional likely pathogenic variant in the *GRID2* gene. Case 19 was compound heterozygous for two missense variants: c.5998 A > G (p.Ile2000Val) and c.963 T > A (p.Asp321Glu), both classified as variant of uncertain significance (VUS).

Two cases (Cases 23 and 24) harbored variants in *SDCCAG8*: a pathogenic frameshift c.1420del (p.Glu474Serfs*20) and a missense VUS c.1489 T > G (p.Phe595Val). Similarly, two cases each were identified with variants in *BBS1* (c.432 + 1G > A and c.1110 + 1G > C), *and BBS7* (c.1083_1084del and c.2015G > T). Finally, one case each was identified with variants in *TTC8* (c.582_585del), *MKS1* (c.515 + 1G > A), *IFT172* (c.4612G > A), *TRIM32* (c.1202_1203insGGAA), and *GRID2* (c.1707_1713del). Notably, Case 5 carried a second homozygous pathogenic variant in *GJB2* (c.299_300delAT; p.H100Rfs*14), consistent with the patient's reported deafness, indicating a possible dual diagnosis.

ACMG classifications of BBS-related variants included *Pathogenic* (15 variants), *Likely Pathogenic* (5 variants), and *VUS* (7 variants). ClinVar entries were available for the majority of previously reported variants, while several novel variants were classified as likely pathogenic or VUS pending further functional studies. All identified variants followed an autosomal recessive inheritance pattern, with homozygous allele read frequencies ranging from 75 to 100%, and population minor allele frequencies (MAF) below 0.01 in gnomAD. Cases 1, 7, 14 and 25 had no report in the ClinVar.

## 4 Discussion

In this study, we performed WES on a cohort of 26 unrelated Iranian patients presenting with clinical features suggestive of BBS. Our analysis identified potentially causal variants in nearly all probands across 13 different genes, confirming the substantial clinical and genetic heterogeneity of BBS. The high detection rate in this cohort, despite the inclusion of cases with atypical or incomplete presentations (e.g., Case 25 with myopathy, Case 22 with encephalocele) shows the practical importance of WES in these types of disorders. In fact, WES can provide a high diagnostic yield and represents an effective first-line or early diagnostic approach in clinically suspected cases of BBS and other genetically heterogeneous ciliopathies, particularly when combined with CNV analysis and applied in consanguineous populations. It is worth mentioning that Case 22 with encephalocele and polycystic kidney disease carrying a homozygous *MKS1* splice-site variant appears to have a phenotype strongly suggestive of a Meckel/ciliopathy spectrum disorder.

The genetic architecture of BBS in our Iranian cohort reveals some distinctions from and similarities to other populations. Globally, *BBS1* (particularly the common c.1169 T > G missense variant (Met390Arg)) is reported as the most frequent causative genes in North American and European cohorts [16]. However, we did not identify any cases with the common *BBS1* Met390Arg variant; instead, we observed two unrelated patients carrying splice site variants in *BBS1* (c.432 + 1G > A and c.1110 + 1G > C). This observation aligns with previous reports suggesting that while *BBS1* Met390Arg is common in outbred populations, its frequency is lower in consanguineous Middle Eastern cohorts, where rarer, family-specific homozygous mutations predominate [20].

The high rate of parental consanguinity (88.4%) in our study is notable. This familial structure greatly increases the likelihood of autosomal recessive disorders and facilitates the identification of large, homozygous loss-of-function variants. Indeed, the majority of our identified variants were homozygous, including several truncating mutations (e.g., *BBS10* c.271dup, *CEP290* c.6869dupA, *TRIM32* c.1202_1203insGGAA). This finding is consistent with the high frequency of consanguineous unions in Iran, leading to the expression of rare, population-specific recessive alleles that might otherwise remain silent.

One interesting observation was the identification of a homozygous exon 6–11 deletion in *BBS2* (Case 5), which is a relatively large structural variant (5.4 Kb). Such copy number variations (CNVs) are often missed by standard WES analysis pipelines that focus primarily on single nucleotide variants and small indels. The successful detection of this deletion highlights the importance of incorporating CNV analysis algorithms into routine WES data interpretation, particularly in highly consanguineous populations where homozygous deletions may be enriched. Case 5 also harbored a second homozygous pathogenic variant in *GJB2* (c.299_300delAT), explaining the patient's deafness—a feature not typically part of the classic BBS phenotype. This incidental finding underscores the power of WES to uncover dual molecular diagnoses in patients with complex or overlapping syndromic features.

Variants of uncertain significance (VUS) were identified in seven cases, most notably in *BBS12* (p.Arg675Gln in two unrelated cases), *BBS7* (p.Gly672Val), *IFT172* (p.Ala1538Thr), and *SDCCAG8* (p.Phe595Val). While these variants meet the criteria for VUS according to ACMG guidelines, their presence in highly conserved residues, homozygous state in affected individuals, absence or extremely low frequency in population databases (gnomAD MAF <0.01), and consistency with the patients' phenotypes suggest they may be reclassified as likely pathogenic upon further functional validation or segregation studies.

The phenotypic spectrum observed in our cohort was broad, ranging from classic BBS presentations to atypical features. The apparently low prevalence of obesity in this cohort (2 out of 26, or 7.7%) should be interpreted cautiously, given the limited availability of systematic anthropometric data. In fact, the absence of a documented diagnosis of obesity was considered evidence of the absence of obesity rather than a systematic approach for assessment of this sign. The discrepancy between our data and other published BBS cohorts -where obesity is found in 50–90% of cases- may be due to a few factors: BMI was not consistently assessed, and obesity was only documented if explicitly noted in clinical records. Many patients were infants or young children at evaluation, so obesity may not have developed yet. The age distribution of our cohort (range: 4 months to 48 years) may have influenced the prevalence of age-dependent features such as obesity and renal disease. Several patients were evaluated during early childhood, before these manifestations typically become apparent. Longitudinal follow-up is needed to capture the full phenotypic spectrum. Additionally, the phenotypic spectrum of BBS may vary between highly consanguineous and outbred populations, potentially due to genetic differences. We recognize this limitation and suggest further longitudinal follow-up to capture age-dependent features. Notably, Case 22 presented with encephalocele and polycystic kidney, a phenotype overlapping with Meckel syndrome, and was found to carry a homozygous splice site variant in *MKS1* (c.515 + 1G > A). This finding reinforces the concept of a ciliopathy spectrum, where mutations in the same gene can give rise to variable phenotypes ranging from severe perinatal lethal conditions (Meckel syndrome) to relatively milder syndromes (BBS or Joubert syndrome), likely modulated by the nature of the mutation and modifier genes [11]. Similarly, Case 18, with a nonsense *CEP290* mutation (p.Gly1890*), exhibited hypotonia and family history without severe renal or neurological involvement, despite *CEP290* mutations typically being associated with Joubert syndrome and Leber congenital amaurosis [26]. Although genotype–phenotype correlations were limited by the small cohort size and lack of longitudinal data, we noted a trend toward more severe ocular and neurological involvement in patients with truncating variants in *BBS10* and *CEP290*, whereas missense variants in *BBS12* and *IFT172* were associated with milder presentations. These observations require validation in larger studies.

This study has several limitations. First, the sample size, while substantial for a rare disease in a single country, is relatively small, limiting the ability to draw robust population-level conclusions about variant frequencies. Second, functional validation (e.g., cellular or animal models) was not performed for the novel or VUS variants. Third, clinical follow-up data were not consistently available, avoiding a detailed genotype-phenotype correlation analysis over time. Forth, clinical features were extracted retrospectively from medical records and information provided by referring clinicians rather than a standardized phenotypic assessment. This should be considered when interpreting the reported frequencies of BBS manifestations. In fact, the relatively low prevalence of obesity and other cardinal BBS features may reflect incomplete phenotypic ascertainment rather than their true prevalence in this cohort. Moreover, age-stratified analysis of the major manifestations was not feasible because of the limited sample size. Finally, a limitation of our study is that parental DNA was not available for all families, preventing segregation analysis in a subset of cases. However, for these families, the candidate variants were supported by homozygosity, rarity, in silico predictions, and phenotypic consistency.

Despite these limitations, the best of our knowledge, this study provides one of the most comprehensive genetic overviews of BBS in the Iranian population, with a relatively large cohort and high diagnostic yield compared to previously published Iranian studies. The findings have several practical implications. For clinical geneticists, this data facilitates evidence-based genetic counseling regarding recurrence risks and prenatal diagnosis options. For the Iranian healthcare system, the identification of recurrent mutations raises the possibility of designing targeted, cost-effective genotyping panels for specific geographic regions or ethnic subgroups. For the broader scientific community, our data adds to the global mutational landscape of BBS, emphasizing the allelic heterogeneity in consanguineous populations and the utility of WES in resolving genetically complex syndromes.

In summary, this study provides one of the most comprehensive genetic overviews of BBS in the Iranian population, identifying a diverse spectrum of BBS-associated variants across 13 genes in 26 families. The most frequently implicated genes were *BBS2*, *BBS5*, and *BBS10*, reflecting the unique genetic architecture of a highly consanguineous population where rare, homozygous loss-of-function variants predominate. The high diagnostic yield of WES underscores its value as a first-line diagnostic tool for clinically suspected BBS and related ciliopathies, particularly when combined with CNV analysis, and supports its early application in patients with atypical or incomplete presentations. The identification of atypical phenotypes and dual molecular diagnoses highlights the phenotypic complexity of ciliopathies and the importance of comprehensive genetic testing to avoid diagnostic delays and misdiagnosis. These findings facilitate accurate genetic counseling, enable carrier screening and prenatal diagnosis in at-risk families, and lay the groundwork for future functional studies and potential gene-based therapies. From a public health perspective, the identification of recurrent mutations in the Iranian population raises the possibility of designing targeted, cost-effective genotyping panels for specific geographic regions or ethnic subgroups. Collectively, our data contribute to the global mutational landscape of BBS, emphasize the importance of population-specific reference databases, and support the integration of WES into routine diagnostic pathways for rare ciliopathies in consanguineous populations.

## Availability of data and materials

The datasets generated and/or analysed during the current study are available in the Clinvar repository (https://www.ncbi.nlm.nih.gov/clinvar/) under accession numbers: VCV000012145.24, VCV001522863.7, and VCV001180799.2.

## CRediT authorship contribution statement

**Sheyda Khalilian:** Investigation. **Mohadeseh Fathi:** Investigation. **Mona Entezam:** Methodology. **Zahra Farbood:** Methodology. **Soudeh Ghafouri-Fard:** Writing – review & editing, Supervision. **Seyed Alireza Dastgheib:** Supervision. **Mohammad Miryounesi:** Supervision.

## Consent of publication

Consent for publication was obtained from patients or their legal representatives.

## Ethics approval and consent to participate

All procedures performed were in accordance with the ethical standards of the institutional and/or national research committee and with the 1964 Helsinki declaration and its later amendments. Informed consent forms were obtained from all patients or their legal representatives. The study protocol was approved by the ethical committee of Shiraz University of Medical Sciences.

## Ethics declaration

Written informed consent to take part in the study and to publish the article has been obtained from all participants or their legal representatives. The privacy rights of participants have been observed.

This study included organ or tissue donors.

This study includes human biological material and consent was obtained by donors, or their next of kin or legal representatives, for use in this study and for publication of the article. The samples used in this research were not sourced from executed prisoners or prisoners of conscience.

This study was performed in compliance with relevant laws, regulatory frameworks and guidelines where the research took place.

This study was approved by the ethical committee of Shiraz University of Medical Sciences.

(Approval No. IR.SUMS.MED.REC.1399.325)

This research follows the CARE guidelines and the CARE checklist.

## Declaration of competing interest

The authors declare they have no conflict of interest.

## Data Availability

No data was used for the research described in the article.
